# Tormentic acid, a triterpenoid isolated from the fruits of *Chaenomeles speciose*, protected indomethacin-induced gastric mucosal lesion via modulating miR-139 and the CXCR4/CXCL12/PLC/PKC/Rho a/MLC pathway

**DOI:** 10.1080/13880209.2023.2249526

**Published:** 2023-08-25

**Authors:** Jun-Yu He, Jie Li, Yuan-Yuan Zhang, Hai-Bo He, Yu-Min He, Dao-Xiang Xu, Xiao Wang, Hao-Yang Wu, Ji-Hong Zhang, Hasan Jahid, Akter Sadia, Hui-Fan Yu, Jun-Zhi Wang, Kun Zou

**Affiliations:** a Department of Clinical Medicine, College of Basic Medical Science, China Three Gorges University, Yichang, P.R. China; b Yichang Key Laboratory of Development and Utilization of Health Products with Drug Food Homology & Hubei Key Laboratory of Natural Products Research and Development, China Three Gorges University, Yichang, P.R. China; c Hubei Key Laboratory of Wudang Local Chinese Medicine Research, Shiyan, P.R. China; d Department of Gastroenterology, Seventh People’s Hospital of Wenzhou, Wenzhou, P.R. China; e Department of Gastroenterology, Chinese Medicine Clinical Medical College & Hubei Clinical Research Center for Functional Digestive Diseases of Traditional Chinese Medicine, China Three Gorges University, Yichang, P.R. China

**Keywords:** Gastric mucosal epithelial cells, cell proliferation and migration

## Abstract

**Context:**

Tormentic acid (TA), an effective triterpenoid isolated from *Chaenomeles speciosa* (Sweet) Nakai (Rosaceae) fruits, exerts an effective treatment for gastric damage.

**Objective:**

To investigate the gastroprotective effect of TA on indomethacin (IND) damaged GES-1 cells and rats, and explore potential mechanisms.

**Materials and methods:**

TA concentrations of 1.563–25 µM were used. Cell proliferation, apoptosis and migration were performed using MTT, colony formation, wound healing, migration, Hoechst staining assays. SD rats were divided into control, IND, TA (1, 2 and 4 mg/kg) + IND groups, once a day for 21 continuous days. Twenty-four hours after the last administration, all groups except the control group were given IND (100 mg/kg) by gavage. Gastric juice parameters, gastric ulcer, gastric blood flow (GBF), blood biochemical parameters and cytokine analysis and gastric mucosal histopathology were detected for 2 h and 6 h after IND oral administration. The mRNA and protein expression of miR-139 and the CXCR4/CXCL12/PLC/PKC/Rho A/MLC pathway were analyzed in the IND-damaged GES-1 cells and gastric tissue of rats.

**Results:**

TA might ameliorate the gastric mucosal injury by accelerating the IND-damaged GES-1 cell proliferation and migration, ameliorating GBF, ulcer area and pathologic changes, the redox system and cytokine levels, the gastric juice parameters, elevating the gastric pH in IND damaged rats; suppressed miR-139 mRNA expression, elevated CXCR4 and CXCL12 mRNA and protein expression, p-PLC, p-PKC, Rho A, MLCK and p-MLC protein expression.

**Discussion and conclusions:**

TA may have potential use as a clinical drug candidate for gastric mucosal lesion treatment.

## Introduction

With the change in modern lifestyles, environmental pollution, and irrational use of non-steroidal anti-inflammatory drugs (NSAIDs), gastrointestinal mucosal lesions have become one of the major diseases that seriously endanger public health and social development, causing great economic and social burdens worldwide, especially in developing countries (O’Malley 2003; Namulema et al. 2018). As gastric mucosal lesion is the main pathophysiological link of gastric ulcers, gastritis, and many other gastric diseases, it is also considered by World Health Organization (WHO) as the inevitable mode of inducing gastric ulcers, chronic gastritis, atrophic gastritis, intestinal metaplasia, atypical hyperplasia, and finally developing into gastric cancer (Da et al. 2019). Thus, there is important clinical urgency to protect gastric mucosa and promote the healing of damaged gastric mucosa for the treatment of gastric diseases and prevention of further deterioration (Aihara et al. 2017). In damaged gastric mucosa, the reconstruction of the gastrointestinal barrier can be mediated by collective migration, proliferation, and subsequent differentiation of epithelial cells (Hoffmann 2005). The process is often achieved by activating the migration of normal epithelial cells around the lesion to the injured area, which can be divided into rapid repair and slow repair: the former refers to the migration of intact epithelial cells around the damaged mucosa to the injured mucosal surface, so that the impaired epithelium can recover continuity and integrity quickly; the latter is the process of cell proliferation that compensates for damaged or necrotic mucosal cells through the cells’ mitosis and differentiation, and the organism restores the damaged gastric mucosal tissue to normal through the inflammatory response, mitotic differentiation of cells, and migration of new cells to cover the damaged area or around the lesion (Liu et al. 2008; Cho and Mills 2019; Engevik et al. 2019). Therefore, the migration and proliferation of gastric epithelial cells are of great significance in promoting the healing of damaged gastric mucosa.

MicroRNAs (miRs) are small non-coding RNA molecules which are capable to down-modulate the translation of target genes by binding to their 3'-untranslated region (3'-UTR). They participate in a wide range of physiological and pathological processes, such as cell cycle, apoptosis, migration, proliferation, differentiation, and angiogenesis (Chen et al. 2018). Recent evidence manifests that miR-139 extensively touched on the pathological processes of digestive system lesions and tumours, Alzheimer’s disease and myocardial injury (Wang et al. 2021). Zhang and his colleagues (2018) indicated that miR-139 might negatively modify CXC motif chemokine receptor 4 (CXCR4) expression, inhibit the migration and proliferation of breast cancer MDA-MB-231 cells by regulating the CXCR4/CXC motif chemokine ligand 12 (CXCL12) signal axis. Simultaneously, our previous studies also identified that the suppressed miR-139 expression effectively activated the CXCR4/CXCL12 pathway to facilitate the migration, proliferation, differentiation of gastric epithelial cells, and the healing of damaged gastric mucosa (Qin et al. 2016a; He et al. 2017b). CXCR4 is known to be a CXCL12-specific receptor consisting of 352 amino acids, which is a protein receptor expressed on different types of cells seven times across the membrane (Hwang et al. 2012). Smith et al. (2005) demonstrated that CXCL12 signalling stimulated epithelial cell migration *via* CXCR4 to reconstruct the mucosal barrier and repair the damaged gastric mucosa. While phospholipase C (PLC) is a target downstream of CXCR4, a class of enzymes existed in the cytoplasmic membrane that can hydrolyze phospholipids, which plays a role in mediating cell signal transduction by triggering the PLC/protein kinase C (PKC) signal cascade (Croitoru et al. 2016). The Ras homolog gene family, member A (Rho A) is a member of the Rho protein family that regulates cell migration, infiltration and metastasis through the reorganization of the cytoskeleton, and is related to cell growth and differentiation by regulating the growth cycle of cells (Rathinam et al. 2011; Cherfils and Zeghouf 2013). Myosin light chain kinase (MLCK) is a serine/threonine protein kinase present in mammals, belongs to the family of calmodulin-dependent protein kinases and participates in many important life activities (Liu et al. 2018). Hwang et al. (2012) and Aihara et al. (2017) found that trifoliate factor may activate CXCR4 in epithelial cells, and then transmit signals and produce effects in sequence by triggering PLC/PKC signal cascade and phosphorylation of amino acid residues. It activates intracellular calcium-dependent signal transduction by increasing the driving force of extracellular Ca^2+^ influx, impels the conversion of Rho A from inactive GDP binding form to active GTP binding form and activates Rho kinase, enhances MLCK activity to stimulate MLC phosphorylation, elevates the formation of actin stress fibers, and induces epithelial cell migration. It could also stimulate E-cadherin relocation and monolayer tightening through Rho-associated protein kinase (ROCK) activation and actin recombination, restore the function of mucosal barrier, and then accelerate the repair of damaged mucosa. With the aforementioned analysis, we can easily discover that miR-139 and the CXCR4/CXCL12/PLC/PKC/Rho A/MLC pathway plays an extremely important role in facilitating the healing of damaged gastric mucosa. Therefore, targeting miR-139 and the CXCR4/CXCL12/PLC/PKC/Rho A/MLC pathway will provide a new therapeutic tool for the prevention and treatment of gastric lesions.

The fruits of *Chaenomeles speciosa* (Sweet) Nakai (Rosaceae), also known as “Mugua”, have the effect of relaxing meridians and activating collaterals, harmonizing stomach, and resolving dampness, which were traditionally used to treat dampness arthralgia, severe pain of waist and knee joint, heat dampness, vomiting and diarrhea, muscle contracture pain, beriberi, edema, etc. (China Pharmacopoeia Committee. 2020). It has been demonstrated that the ethyl acetate extract of *Chaenomeles speciosa* fruits and its active pentacyclic triterpenoid constituents exhibited good therapeutic effects on gastric ulcer patients and animals (Rodríguez et al. 2003; Nam et al. 2006; Qin et al. 2015, 2016b; He et al. 2017a). A large amount of research indicates that pentacyclic triterpenoids have a wide range of biological activities, such as anti-inflammatory, antioxidant activities and gastric mucosal protective effects, whereas the activity of single pentacyclic triterpenoids (such us oleanolic acid) is mainly anti-inflammatory and antioxidant (Ishikawa et al. 2008). With an increase in the numbers or types of pentacyclic triterpenoids [e.g., niga-ichigoside F1, 23-hydroxy formic acid, oleanolic acid, ursolic acid, 3-*O*-acetyl ursolic acid, betulinic acid, 3-*O*-acetyl pomolic acid, maslinic acid, and tormentic acid (TA)], their gastroprotective effects were significantly enhanced compared with single pentacyclic triterpenoids, but their anti-inflammatory and antioxidant activities were retained (Gomes et al. 2009; Qin et al. 2016a; He et al. 2017b; da Rosa et al. 2018; Pan et al. 2018). Some pentacyclic triterpenoids were also extracted and isolated from *Chaenomeles speciosa* fruits in our previous study (Shi et al. 2016), However, little is known about the pharmacological activity and mechanism of each triterpene component. Therefore, in the current study, we first screened the triterpenoid compound with the best gastroprotective activity among the pentacyclic triterpenoids from *Chaenomeles speciosa* fruits, and then further investigated the gastroprotective effects of the most active triterpenoid on IND-damaged human gastric mucosal epithelial cells (GES-1) and rats and potential mechanisms through modulating miR-139-mediated CXCR4/CXCL12/PLC/PKC/Rho A/MLC pathway.

## Materials and methods

### Experimental material

The fruits of *Chaenomeles speciosa* were purchased from Langping Mugua Planting Base (Yichang, China) on 18 July 2021, and identified by Professor Yubing Wang. The voucher specimens (2021-0745) are preserved in Yichang Key Laboratory of Development and Utilization of Health Products with Drug Food Homology, China Three Gorges University. Oleanolic acid, ursolic acid, 3-*O*-acetyl ursolic acid, betulinic acid, 3-*O*-acetyl pomolic acid, maslinic acid, and TA used in this study were isolated from *Chaenomeles speciosa* fruits as previously described (Shi et al. 2016). The aforementioned isolated compounds were analyzed by liquid chromatography-mass spectrometry and found to be over 95% pure.

### Reagents

RPMI-1640 medium, phosphate-buffered saline (PBS), fetal bovine serum (FBS), trypsin, penicillin and streptomycin were obtained from Gibco Company (Carlsbad CA, USA). Lactate dehydrogenase (LDH), total antioxidant capacity (T-AOC), superoxide dismutase (SOD), glutathione peroxidase (GSH-Px), catalase (CAT), xanthine oxide (XOD), and malondialdehyde (MDA) were purchased from Nanjing Jiancheng Biologic Engineering Institute (Nanjing, China). Tumour Necrosis Factor-α (TNF-α), interleukin-1β (IL-1β), IL-6, IL-10, and IL-4 ELISA kits were all purchased from Biolegend, Inc. (San Diego, CA, USA). Dimethyl sulfoxide (DMSO), indomethacin (IND), crystal violet, Hoechst 33258, 3-(4,5-dimethylthiazol-2-yl)-2,5-diphenyltetrazolium bromide (MTT), and 5,5',6,6'-tetrachloro-1,1',3,3'-tetraethylben-zimidazolcarbocyanine iodide (Mitochondrial membrane potential detection kit, JC-1) were purchased from Sigma-Aldrich (St. Louis, MO, USA). Annexin V-FITC/Propidium Iodide (PI) apoptotic detection kit was obtained from BD Biosciences (Bedford, MA, USA). EasyScript First-Strand cDNA Synthesis SuperMix, SYBR Green real-time PCR Master Mix reagents and Dual Luciferase Reporter Assay System were purchased from Promega Corporation (Madison, WI, USA). Trizol isolation kit was obtained from Invitrogen Corporation (Carlsbad, CA, USA). miR-139 mimic, miR-139 inhibitor, miR mimic negative control (mimic NC), inhibitor negative control (inhibitor NC), siRNA of CXCR4 (CXCR4 siRNA) and negative siRNA (siRNA NC) were obtained from GenePharma Company (Shanghai, China). Lipofectamine 3000 reagent was purchased from Invitrogen Corporation (Carlsbad, CA, USA). Primary antibodies (CXCR4, CXCL12, PLC, phosphorylated phospholipase C (p-PLC), PKC, phosphorylated protein kinase C (p-PKC), Rho A, MLCK, MLC, phosphorylated myosin light chain (p-MLC) and secondary antibodies were purchased from Cell Signaling Technology (Danvers, MA, USA). Western Bright ECL prime detection reagent, SDS-PAGE gel kit, and PVDF membrane were obtained from Boshide Biotechnology Company (Wuhan, China). All other regents used in the present study were of analytical grade.

### Cell culture and transfection

GES-1 cells (CRL-1040) were obtained from the American Type Culture Collection and grown in RPMI-1640 medium containing 10% FBS and 1% antibiotic (100 μg/mL streptomycin and 100 units/mL penicillin) at 37 °C in a humidified 5% CO_2_ atmosphere, which was seeded in a 6-well plate to adhere for 24 h, and then GES-1 cells were transfected miR-139 mimic, miR-139 inhibitor, CXCR4 siRNA or scrambled miR NC for 24 h. The detailed methods were carried out according to our previous description (He et al. 2020).

### Viability assay

Cellular proliferative viability was detected using the MTT assay (Qin et al. 2016a). Briefly, GES-1 cells (1 × 10^5^ cells/well) were seeded in a 96-well plate to adhere for 24 h, and then pretreated with oleanolic acid, ursolic acid, 3-*O*-acetyl ursolic acid, betulinic acid, 3-*O*-acetyl pomolic acid, maslinic acid, and TA (1.563, 3.125, 6.25, 12.5, 25, and 50 µM) for 6 h, then continued or treated with IND in the concentration of 700 μM for 18 h. Next MTT solution (20 µL, 5 mg/mL) was added to each well and incubated for 4 h at 37 °C. Then the medium was discarded and 150 mL DMSO was added to dissolve the formazan crystals. Finally, viable cells were detected by measuring absorbance at 570 nm using a Microplate Reader (Tecan, Switzerland). The LD_50_ value, the drug concentration causing 50% cell death, was estimated from survival curves using the logit method. The cell viability assays of GES-1 cells pretreated with TA (12.5 μM) used alone or combined with miR-139 mimic, miR-139 inhibitor, and CXCR4 siRNA were also performed according to this method.

### LDH leakage assay

The GES-1 cells (300 cells/well) were seeded in a 6-well plate to adhere for 24 h, and the treatments of GES-1 cells were similar to “Viability assay”. After the end of the culture, the supernatant of each well was collected, and the LDH leakage was determined using an assay kit according to our previous description (He et al. 2020). The LDH leakage assays of GES-1 cells pretreated with TA (12.5 μM) used alone or combined with miR-139 mimic, miR-139 inhibitor, CXCR4 siRNA were detected in a similar manner.

### Colony formation assay

The adherent GES-1 cells (300 cells/well) seeded in 6-well plates were treated with TA (12.5 μM) used alone or combined with the miR-139 mimics, miR-139 inhibitor and CXCR4 siRNA. The medium was changed every 3 days. 18 h before the end of the experiment, the concentration of 700 μM IND was added to each group except the control group. After 14 days of culture, the cells were collected, stained with 0.1% crystal violet, and washed with PBS to remove the residual dye, and then the clone numbers were counted and photographed under a microscope (Olympus, Tokyo, Japan).

### Wound healing assay

After the GES-1 cells (1 × 10^5^ cells/well) were seeded in 24-well plates to adhere for 24 h, the cells divided into TA (12.5 μM) and transfection with miR-139 mimic, miR-139 inhibitor, CXCR4 siRNA or in combination with TA groups, and scratched with a 200-µL micropipette tip to create a uniform wound. After washing away the floating cells with PBS, TA (12.5 μM) was added, and continued to culture for 6 h, added 700 μM IND except for the control group at 6-h intervals, and then continuously cultured. Phase contrast images were noted (0, 12, and 24 h after scratching) under a microscope (Olympus, Tokyo, Japan), and analyzed using Image J software system (Bethesda, MD, USA).

### Migration assay

The GES-1 cells transfected with miR-139 mimic, miR-139 inhibitor and CXCR4 siRNA during the logarithmic growth phase were subjected to routine digestion and centrifugation, and the cells were resuspended in serum-free 1640 medium to prepare 5 × 10^5^ cells/mL cell suspension, and 100 μL cell suspension was plated on the upper chamber of the insert; the lower chamber was filled with 10% FBS medium which contained IND (700 μM) and/or TA (12.5 μM) as the control group, IND group, TA (12.5 μM) groups, respectively. After cultured in a 37 °C incubator for 24 h, the upper chamber was taken out and rinsed twice with PBS, fixed by methanol, stained with 0.1% crystal violet, and then observed and photographed under a microscope (Olympus, Tokyo, Japan) at 100 × magnification. Cells from five random fields per filter were counted using Image J software system (Bethesda, MD, USA).

### Hoechst staining

GES-1 cells (1 × 10^5^ cells/well) were seeded in 6-well plates to adhere for 24 h, after pretreatment with TA (12.5 μM) used alone or in combination with the miR-139 mimics, miR-139 inhibitor and CXCR4 siRNA for 6 h, 700 μM IND was added except for the control group. After 18 h of continuous culture, Hoechst staining was performed according to our previous description (He et al. 2020).

### Annexin V-FITC/PI cytometric assay

GES-1 cells (1 × 10^5^ cells/well) were inoculated into 6-well plates for 24 h after adhesion, and the cells were pretreated with TA (12.5 μM) used alone or combined with the miR-139 mimics, miR-139 inhibitor and CXCR4 siRNA for 6 h, then added 700 μM IND except for the control group. After 18 h of continuous incubation, the cells were collected and stained with Annexin V-FITC/PI detection kit. Apoptosis was measured by flow cytometer (BD Biosciences, MA, USA).

### Mitochondrial viability assay

The adherent GES-1 cells were pretreated with TA (12.5 μM) used alone or in conjunction with the miR-139 mimics, miR-139 inhibitor and CXCR4 siRNA for 6 h, and then added 700 μM IND except for the control group. After 18 h of continuous culture, the cells were collected, and the mitochondria viability was detected according to the method introduced in our previous description (Qin et al. 2016a).

### Luciferase reporter assay

The predicted binding site in the 3'-UTR of CXCR4 was elaborated by PCR, and inserted into the pmir GLO Luciferase miRNA Target Expression Vector (Promega, Madison, WI, USA), which was known as CXCR4 wild type (CXCR4-WT). The predicted binding sites were substituted for forming the negative control: CXCR4 mutated type (CXCR4-MUT). The vectors were transfected with miR-139 mimic or the mimic NCs with Lipofectamine 3000 reagent. After 48 h of transfection, the luciferase activities were detected with the Luciferase Reporter Assay System (Promega, Madison, WI, USA) according to the manufacturer’s instructions.

### Establishment of rat gastric mucosal damage model and treatments

Male Sprague-Dawley rats (6 weeks old) supplied by the Laboratory Animal Center of China Three Gorges University (Yichang, China), were used for this study. The experimental rats were housed under a 12 h light/dark cycle and temperature and humidity-controlled environment, and food and water were supplied *ad libitum*, the welfare and experimental procedures were carried out following the National Institutes of Health (Bethesda, MD, USA) and the related ethical regulations of China Three Gorges University. The experimental protocols were agreed upon by the Animal Ethics and Welfare Committee of China Three Gorges University (Permission number: CTGUAEWC-2021-079).

The rat model of the gastric mucosal lesion was established according to our previous description (Qin et al. 2016a). Briefly, the rats were randomized into five groups with 20 rats in each group including control, IND, TA-L (1 mg/kg) + IND, TA-M (2 mg/kg) + IND and TA-H (4 mg/kg) + IND groups after a week of adaptation. The experimental rats in each TA group were administrated previously with TA once a day for 21 continuous days, and rats in the control group and IND group were administrated with a vehicle (0.5% carboxymethylcellulose sodium) of the same volume. After the last administration, the rats were deprived of food but allowed to drink freely. After 24 h, all groups except the control group were given IND (100 mg/kg) by gavage. The experiment was implemented 2 h and 6 h after IND oral administration, respectively.

### Gastric juice parameter determination

Two h after IND oral administration, the pylorus ligation of 10 rats randomized from each experimental group was carried out under pentobarbital sodium (50 mg/kg, *i.p.*) anaesthesia, meanwhile, the drinking water was also deprived. After pylorus ligation for 4 h, rats were sacrificed, and the stomachs were removed and cut open along the greater curvature of the stomach, the gastric contents were collected. The gastric juice parameters (volume, pH, and total acidity) were detected as previously described by Dhiyaaldeen et al. (2014).

### Gastric ulcer analysis

After collecting gastric contents, the stomachs were flushed with ice normal saline, and then photographed. The ulcer area and inhibition rate were calculated according to our previous description (He et al. 2020). After taking pictures of the stomachs, the gastric tissue samples selected from each stomach were soaked in 4% paraformaldehyde for pathologic examination, and the residual gastric tissues were preserved at −80 °C for biochemical and molecular analyses.

### GBF detection

Six hours after IND oral administration, the surplus rats per group were anaesthetized with pentobarbital sodium, and the stomachs were opened along the greater curvature of the stomach for the gastric blood flow (GBF) detection at ulcer margin by the Laser Doppler Flowmeter (Vasamedics, St. Paul, MN, USA) as previously described by Magierowski et al. (2017).

### Gastric wall mucus content measurement

After detecting GBF, the blood samples of experimental rats were collected and sacrificed, and the stomach was removed. The mucus contents of the gastric wall were determined according to the method described by Dhiyaaldeen et al. (2014).

### Biochemical analysis

The collected blood samples were centrifuged at 3000 rpm at 4 °C for 15 min. Supernatant serums were transferred to clean EP tubes and stored at −80 °C. The T-AOC, SOD, GSH-Px, CAT, XOD, and MDA levels in serum were measured by chemichromatometry according to the directions of the reagent kits.

### Cytokine assay

The contents of TNF-α, IL-6, IL-1β, IL-10, and IL-4 were detected as the instructions of the kit in the gastric mucosa tissues of experimental rats.

### Histopathology analysis

Gastric tissues were fixed in 4% paraformaldehyde, sectioned, and then stained with hematoxylin and eosin (H&E). The sections were evaluated according to the criteria established by Laine and Weinstein (1988), and comments were blind.

### Quantitative real-time polymerase chain reaction (qRT-PCR) analysis

Total RNAs were extracted from GES-1 cells and gastric tissue using Trizol kit (Invitrogen, Carlsbad, CA, USA) and reversed transcribed into cDNAs. miRNA and mRNA expression levels were quantified by using qPCR with SYBR Green real-time PCR Master Mix kits. U6 and GAPDH acted as the internal controls for miRNAs and mRNA, respectively. The primer sequences used in qRT-PCR are listed in Table 1, and synthesized by Shanghai Shenggong Bioengineering Co., Ltd. (Shanghai, China). The comparative quantitative and statistical analyses were implemented using the 2^-ΔΔCt^ method.

**Table 1.** Primer sequences used in qRT-PCR.

Genes	Forward primer (5′-3′)	Reverse primer (5′-3′)
hsa-miR-139	ACACTCCAGCTGGGTCTACAGTGCACGTGTC	TGGTGTCGTGGAGTCG
hCXCR4	TGGCCTTATCCTGCCTGGTAT	GGAGTCGATGCTGATCCCAAT
hCXCL12	CTCAACACTCCAAACTGTGCCC	CTCCAGGTACTCCTGAATCCAC
rno-miR-139	ACACTCCAGCTGGGTCTACAGTGCAC	TGGTGTCGTGGAGTCG
rCXCR4	CGAGCATTGCCATGGAAATA	CGGAAGCAGGGTTCCTTGT
rCXCL12	AGCCAACGTCAAACATCTGAAA	CGGGTCAATGCACACTTGTC
U6	GATCCCTCCAAAATCAAGTGG	GGCAGAGATGATGACCCTTTT
GAPDH	GAAGGTGAAGGTCGGAGTC	GAAGATGGTGATGGGATTTG

### Western blotting analysis

The total proteins of the GES-1 cells and gastric tissues were extracted by using protein extraction kits. The protein content was measured by a nucleic acid protein analyzer (Thermo Scientific, USA). Cellular and tissue proteins (50 μg) were transferred to the PVDF membrane, and the blots were probed with primary antibodies against CXCR4, CXCL12, PLC, p-PLC, PKC, p-PKC, Rho A, MLCK, MLC, p-MLC, and β-actin at 4 °C overnight and then incubated with secondary antibody. The blots were developed using an ECL detection kit. Quantitative analysis was executed by using Image J Morphology Analysis System (National Institute of Health, USA), and molecular expression was normalized to β-actin.

### Statistical analysis

The results were expressed as mean ± SD. Data were processed and analyzed using SPSS 21.0 software, Image-proplus 6.0, GraphPad Prism 7.0, and One-way Analysis of Variance (ANOVA) was used for multi-group comparison. Differences were considered statistically significant at *P* values less than 0.05.

## Results

### Selection of TA based on the cytoprotective effect on IND-damaged GES-1 cells

**Figure 1.**
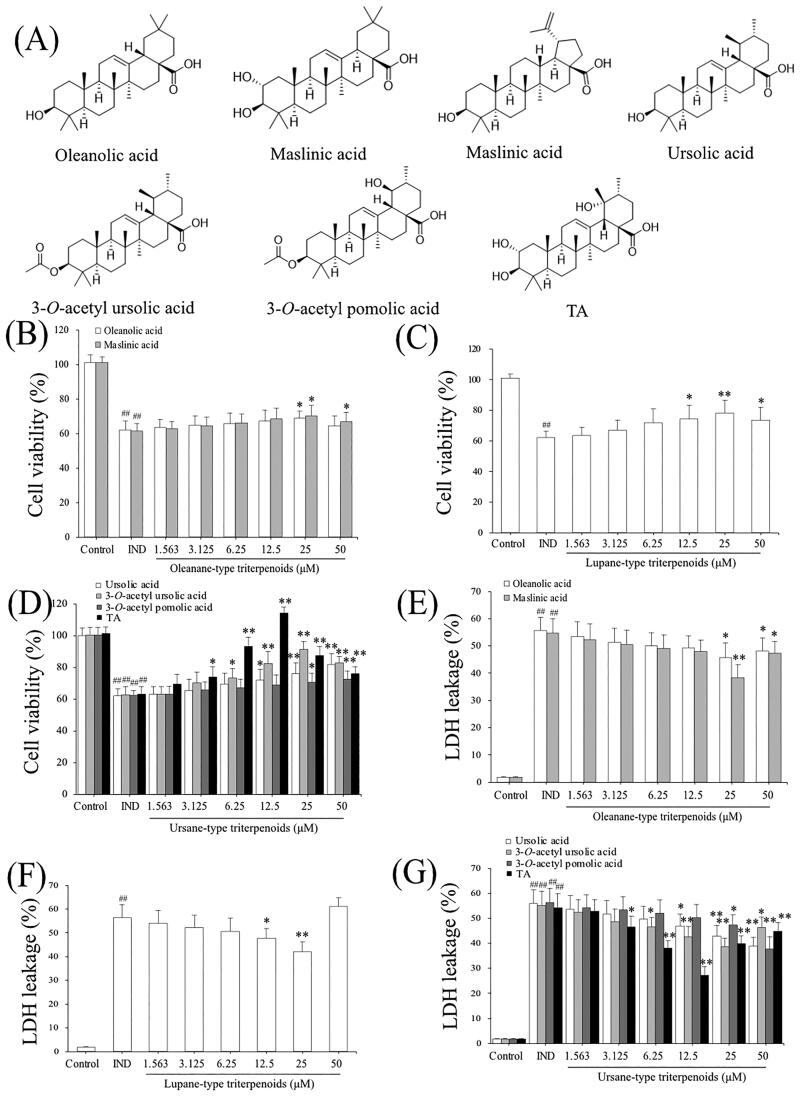
Chemical structures of the pentacyclic triterpenoids (oleanolic acid, ursolic acid, 3-*O*-acetyl ursolic acid, betulinic acid, 3-*O*-acetyl pomolic acid, maslinic acid and TA) from *Chaenomeles speciosa* fruits, their effects on IND-damaged cell proliferation and suppressed LDH leakage. (A) Chemical structures of the pentacyclic triterpenoids. (B) Cell viability of oleanane-type triterpenoids. (C) Cell viability of lupane-type triterpenoids. (D) Cell viability of ursane-type triterpenoids. (E) LDH leakage of oleanane-type triterpenoids. (F) LDH leakage of lupane-type triterpenoids. (G) LDH leakage of ursane-type triterpenoids. The data are indicated as the means ± SD (*n* = 5). ^#^*p* < 0.05, ^##^*p* < 0.01 compared to the control group; *p < 0.05, **p < 0.01 compared to the IND group.

The structures of pentacyclic triterpenoids (oleanolic acid, maslinic acid, betulinic acid, ursolic acid, 3-*O*-acetyl ursolic acid, 3-*O*-acetyl pomolic acid, and TA) from the fruits of *Chaenomeles speciosa* are shown in Figure 1(A). The effects of the aforementioned pentacyclic triterpenoids on GES-1 cell proliferation *in vitro* were tested using the MTT assay. Following treatment with 1.563-50 μM of the aforementioned pentacyclic triterpenoids for 24 h, oleanolic acid (25 μM), maslinic acid (25 μM), betulinic acid (12.5, 25 μM), ursolic acid (12.5, 25, 50 μM), 3-*O*-acetyl ursolic acid (6.25, 12.5, 25 μM), 3-*O*-acetyl pomolic acid (25, 50 μM) and TA (3.125, 6.25, 12.5 μM) significantly promoted the IND-damaged cell proliferation compared to the IND-damaged GES-1 cell group (*p*** **<** **0.05 or *p*** **<** **0.01, respectively, Figure 1(B–D)). The ED_50_ and LD_50_ values were 7.16, 10.65, 5.80, 6.46, 11.58, 7.07, 3.02 μM and 180.93, 235.26, 158.98, 190.76, 144.41, 216.91, 95.41 μM in IND-damaged GES-1 cells treated with oleanolic acid, maslinic acid, betulinic acid, ursolic acid, 3-*O*-acetyl ursolic acid, 3-*O*-acetyl pomolic acid, and TA, respectively, for 24 h. The cell proliferation indexes (TI) were 25.27, 22.09, 27.41, 29.53, 12.47, 30.68, 31.59, respectively. These results indicated that TA had a better proliferative activity of IND-damaged GES-1 cells than the other pentacyclic triterpenoids.

Cell lesion or death was also quantitatively evaluated by detecting LDH leakage from the damaged cells; the more injured a cell is, the more it leaks. So, its leakage rate from the cytoplasm into the medium is an important indicator of verifying the integrity of the cell membrane and the degree of cell damage (Wang et al. 2019). To further investigate the protective effects of the pentacyclic triterpenoids (oleanolic acid, maslinic acid, betulinic acid, ursolic acid, 3-*O*-acetyl ursolic acid, 3-*O*-acetyl pomolic acid, and TA), the LDH release rates under their intervention were evaluated. As indicated in Figure 1(E–G), LDH leakage was elevated 30-fold in the IND-damaged group compared to the control group (*p*** **<** **0.01). In contrast, oleanolic acid (25 μM), maslinic acid (25 μM), betulinic acid (12.5, 25 μM), ursolic acid (12.5, 25, 50 μM), 3-*O*-acetyl ursolic acid (6.25, 12.5, 25 μM), 3-*O*-acetyl pomolic acid (25, 50 μM) and TA (3.125, 6.25, 12.5 μM) treatments prominently restrained the IND-damaged cell LDH leakage compared to IND group (*p*** **<** **0.05 or *p*** **<** **0.01, respectively). Based on these studies, TA among the pentacyclic triterpenes from *Chaenomeles speciosa* fruits and its appropriate concentrations (under 12.5 μM), was selected for further study.

### TA promoted IND-damaged GES-1 cell proliferation and restrained apoptosis

**Figure 2.**
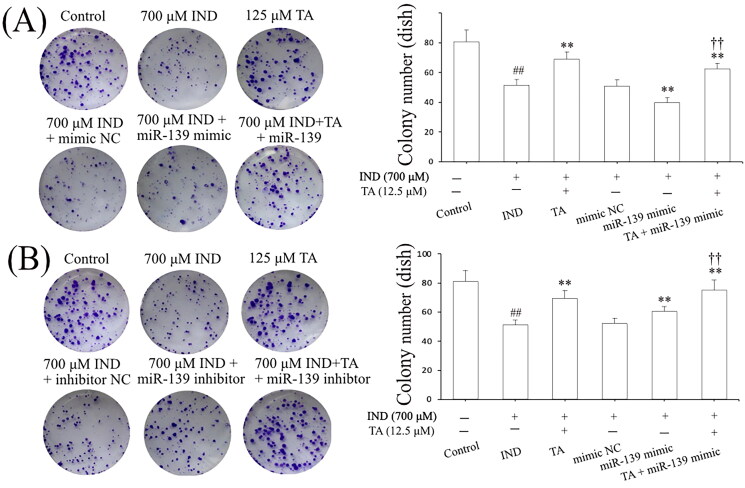
TA promoted IND-damaged GES-1 cell proliferation. (A) Cell proliferation by colony formation analysis of IND-damaged GES-1 cells in the pretreatment with TA used alone or combined with the miR-139 mimics. (B) Cell proliferation by colony formation analysis of IND-damaged GES-1 cells in the pretreatment with TA used alone or combined with the miR-139 inhibitor. The data are indicated as the mean ± SD (*n* = 5). ^#^*p* < 0.05, ^##^*p* < 0.01 compared to the control group; *p < 0.05, **p < 0.01 compared to the IND group; ^†^*p* < 0.05, ^††^*p* < 0.01 compared to the mimic NC or inhibitor NC group.

**Figure 3.**
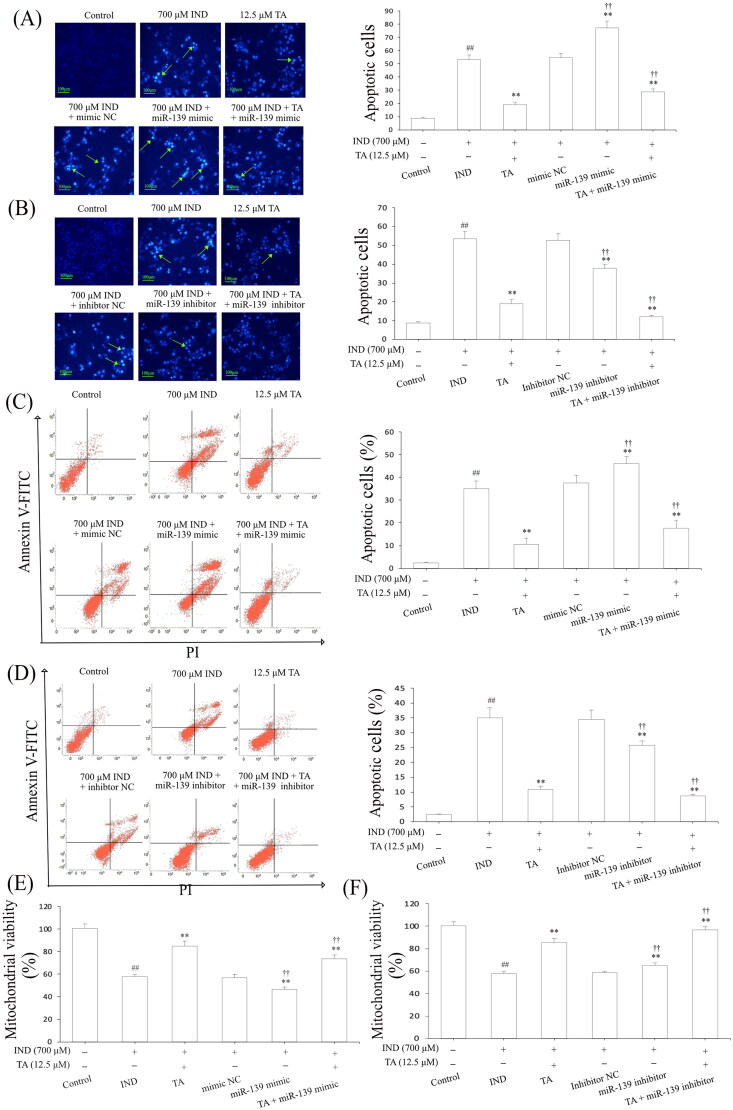
TA restrained IND-damaged GES-1 cell apoptosis. (A) and (B) Apoptotic cells by Hoechst-staining analysis. (C) and (D) Apoptotic cells by flow cytometry analysis. (E) and (F) Mitochondrial viability by flow cytometry analysis. The IND-damaged GES-1 cells were pretreated with TA used alone or combined with the miR-139 mimics and miR-139 inhibitor. Typical apoptotic cells were demonstrated by a green arrowhead. The data are indicated as the mean ± SD (*n* = 5). ^#^*p* < 0.05, ^##^*p* < 0.01 compared to the control group; *p < 0.05, **p < 0.01 compared to the IND group; ^†^*p* < 0.05, ^††^*p* < 0.01 compared to the mimic NC or inhibitor NC group.

Niv and Banić (2014) demonstrated that inhibition of apoptosis and promotion of proliferation of gastric mucosal epithelial cells were a prerequisite for promoting the repair of damaged gastric mucosa. After assessing the influence of the pentacyclic triterpenoids and the safe concentration range of TA, we further investigated its effects on the apoptosis and proliferation of IND-damaged GES-1 cells in the pretreatment with TA used alone or combined with the miR-139 mimics and miR-139 inhibitor. As indicated in Figure 2(A) and Figure 3(A, C and E), the proliferative activities of the IND-damaged or combined transfection with miR-139 mimic GES-1 cells were dramatically suppressed, mitochondrial viabilities were reduced, and apoptosis was elevated compared to the control or mimic NC group (*p*** **<** **0.01, respectively). TA used alone or combined with the miR-139 mimics obviously promoted IND-damaged GES-1 cell proliferation, raised mitochondrial viabilities and repressed apoptosis compared to the IND or mimic NC group (*p*** **<** **0.01, respectively). Interestingly, transfection with miR-139 inhibitor, TA used alone or in combination with TA dramatically accelerated IND-damaged GES-1 cell proliferation, elevated mitochondrial viabilities and suppressed apoptosis compared to the IND or inhibitor NC group (*p*** **<** **0.01, respectively, Figure 2(B) and Figure 3(B, D and F)). The aforementioned data indicated that the pro-proliferative and anti-apoptotic effects of TA were related to suppressing miR-139 expression.

### TA enhanced IND-damaged GES-1 cell migration

**Figure 4.**
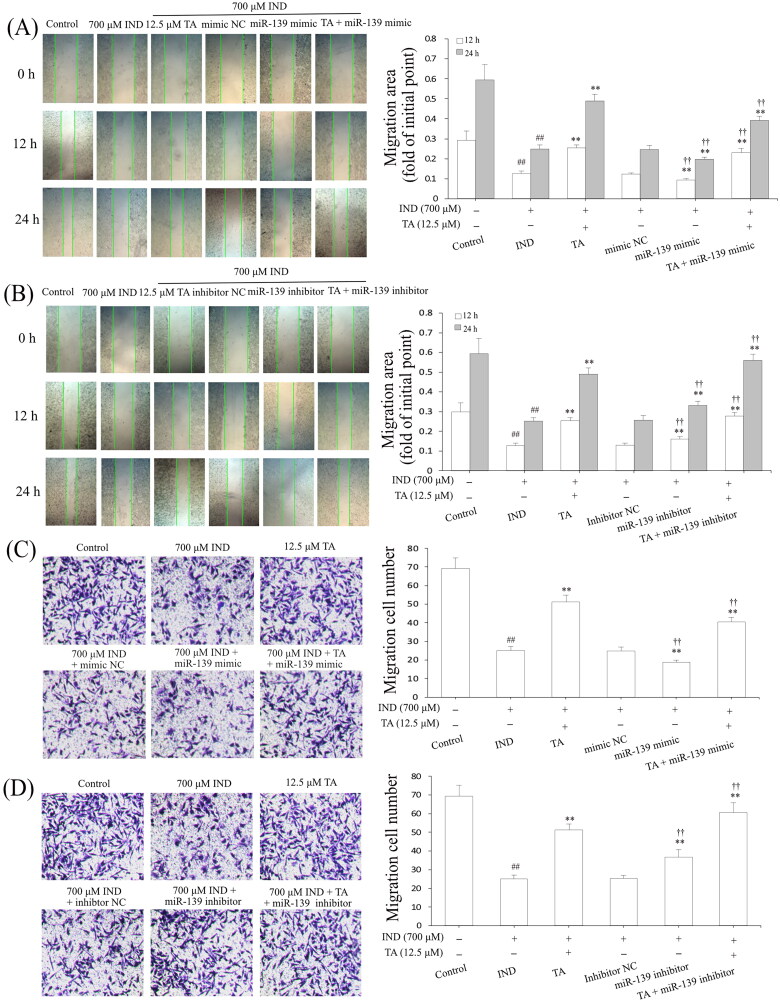
TA enhanced IND-damaged GES-1 cell migration. (A) and (B) Cell migration by wound healing assay. (C) and (D) Cell migration by Transwell assay. The IND-damaged GES-1 cells were pretreated with TA used alone or combined with the miR-139 mimics and miR-139 inhibitor. The data are indicated as the mean ± SD (*n* = 5). ^#^*p* < 0.05, ^##^*p* < 0.01 compared to the control group; *p < 0.05, **p < 0.01 compared to the IND group; ^†^*p* < 0.05, ^††^*p* < 0.01 compared to the mimic NC or inhibitor NC group.

The gastric epithelium, an important part of the gastric mucosal barrier, plays a critical role in repairing the damaged gastric mucosa and maintaining mucosal structural integrity. After gastric mucosal damage, the migration and proliferation of adjacent gastric mucosal epithelial cells are an important way to complete this process (Aihara et al. 2018). After accomplishing the analysis of TA promoting GES-1 cell proliferation, we further studied its effect on IND-damaged GES-1 cell migration by wound healing and Transwell assays, respectively. As demonstrated in Figure 4(A and C), the migration activity of the IND-damaged or combined with the miR-139 mimics GES-1 cells was significantly depressed compared to the control or mimic NC group (*p*** **<** **0.01, respectively). TA used alone or combined with the miR-139 mimics obviously accelerated IND-damaged GES-1 cell migration compared to the IND or mimic NC group (*p*** **<** **0.01, respectively). In contrast, transfection with miR-139 inhibitor or combination with TA or TA used alone prominently enhanced IND-damaged GES-1 cell migration compared to the IND or inhibitor NC group (*p*** **<** **0.01, respectively, Figure 4(B–D)). The data suggested a potential migration-promoting activity of TA, which was a relation to repressing miR-139 expression in IND-damaged GES-1 cells.

### TA facilitated the IND-damaged GES-1 cell migration via suppressing miR-139 expression and activating the CXCR4/CXCL12/PLC/PKC/Rho a/MLC pathway

**Figure 5.**
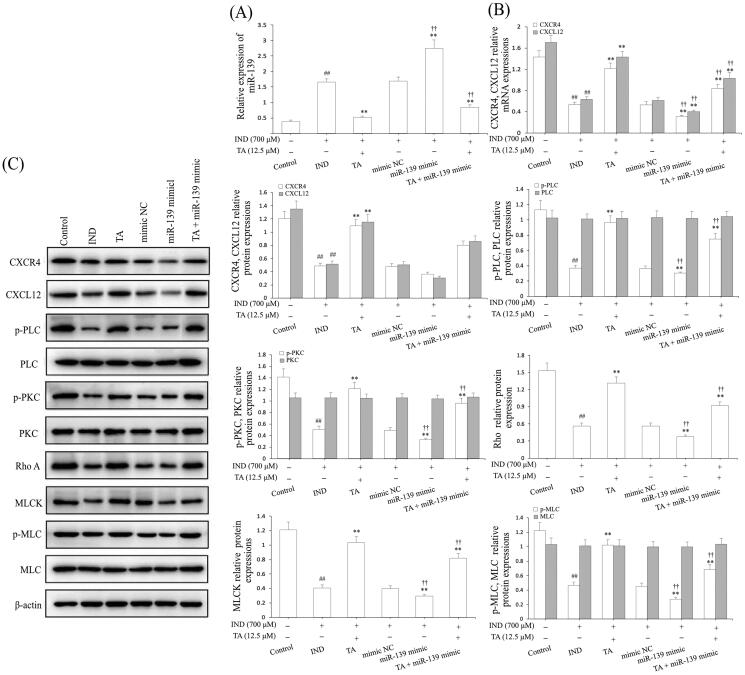
TA facilitated IND-damaged GES-1 cell migration *via* downregulating miR-139 and activating the CXCR4/CXCL12/PLC/PKC/Rho a/MLC pathway. (A) miR-139 mRNA expression. (B) CXCR4 and CXCL12 mRNA expression. (C) Related protein expression of the CXCR4/CXCL12/PLC/PKC/Rho a/MLC pathway. The IND-damaged GES-1 cells were pretreated with TA used alone or combined with the miR-139 mimics. The data are indicated as the mean ± SD (*n* = 5). ^#^*p* < 0.05, ^##^*p* < 0.01 compared to the control group; *p < 0.05, **p < 0.01 compared to the IND group; ^†^*p* < 0.05, ^††^*p* < 0.01 compared to the mimic NC group.

The above results demonstrated the protective effect of TA was related to promoting IND-damaged GES-1 cell migration. To confirm whether miR-139 was involved in the migration effects of TA on IND-damaged GES-1 cells, we analyzed miR-139 expression. As illustrated in Figure 5(A), the miR-139 mRNA expression in IND-damaged GES-1 cells was notably elevated compared to the control group (*p*** **<** **0.01), and the mRNA expression level of miR-139 was further elevated in the miR-139 mimic-transfected GES-1 cells compared to the mimic NC group (*p*** **<** **0.01). Instead, IND-damaged and miR-139 mimic-transfected IND-damaged GES-1 cells pretreated with TA (12.5 μM) exhibited dramatically reduced miR-139 expression compared to the IND and mimic NC groups (*p*** **<** **0.01, respectively). The current results are consistent with TA accelerating IND-damaged GES-1 cell migration in wound healing and Transwell assays (Figure 4(A and C)), which demonstrated that the cytoprotective and migration-promoting effects of TA were related to restraining miR-139 expression.

Hwang et al. (2012) and Aihara et al. (2017) found that the CXCR4/CXCL12/PLC/PKC/Rho A/MLC pathway played an extremely important role in ameliorating the damaged gastric mucosal repair and healing. To illustrate this, the protein expression assay of this pathway was performed in the miR-139 mimic-transfected IND-damaged GES-1 cells. As illustrated in Figure 5(B and C), the CXCR4, CXCL12 mRNA and protein expression and p-PLC, p-PKC, Rho A, MLCK and p-MLC protein expression in IND-damaged GES-1 cells was dramatically reduced compared to the control group (*p*** **<** **0.01). After the IND-damaged GES-1 cells were transfected with miR-139 mimic, the above-mentioned protein expression was revised down sharply compared to the mimic NC group (*p*** **<** **0.01). In contrast, TA (12.5 μM) used alone or combined with the miR-139 mimics markedly elevated CXCR4, CXCL12 mRNA and protein expression and p-PLC, p-PKC, Rho A, MLCK and p-MLC protein expression levels compared to the IND or mimic NC group (*p*** **<** **0.01, respectively). There were no obvious changes in the protein expression of PLC, PKC and MLC after administration of TA used alone or in combination with miR-139 mimic.

### miR-139 knockdown reinforced the activating effect of TA on the CXCR4/CXCL12/PLC/PKC/Rho a/MLC signaling pathway by targeting the CXCR4 gene

**Figure 6.**
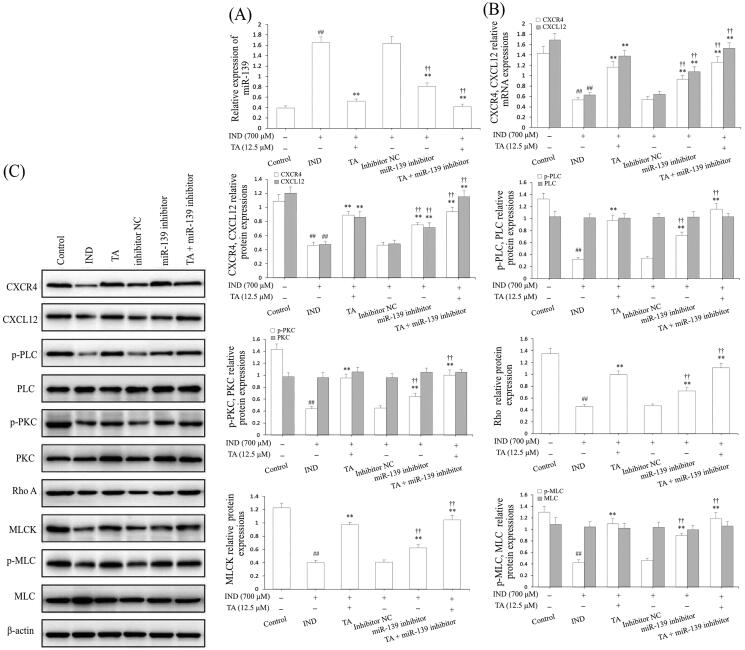
miR-139 knockdown reinforced the accelerating effect of TA on CXCR4/CXCL12/PLC/PKC/Rho A/MLC pathway. (A) miR-139 mRNA expression. (B) CXCR4 and CXCL12 mRNA expression. (C) Related protein expression of the CXCR4/CXCL12/PLC/PKC/Rho a/MLC pathway. The IND-damaged GES-1 cells were pretreated with TA used alone or combined with the miR-139 inhibitor. The data are indicated as the mean ± SD (*n* = 5). ^#^*p* < 0.05, ^##^*p* < 0.01 compared to the control group; *p < 0.05, **p < 0.01 compared to the IND group; ^†^*p* < 0.05, ^††^*p* < 0.01 compared to the inhibitor NC group.

To further validate whether miR-139 was extended to IND-damaged GES-1 cell migration, we analyzed GES-1 cell viability, apoptosis and detected migration, miR-139 expression and the CXCR4/CXCL12/PLC/PKC/Rho A/MLC pathway related protein expression after the IND-damaged GES-1 cells were deleted of the miR-139 gene by transfecting with miR-139 inhibitor or combined with TA or TA used alone. As Figure 2(B), Figure 3(B–F), Figure 4(B and D) and Figure 6(A) indicate, the knockdown of miR-139, TA used alone or combined with TA significantly facilitated cell migration, proliferation, raised mitochondrial viability, reduced apoptosis and miR-139 expression in the IND-damaged cells or miR-139 inhibitor-transfected IND-damaged GES-1 cells compared to the IND or inhibitor NC group. Simultaneously, western blotting results also indicated that the CXCR4/CXCL12/PLC/PKC/Rho A/MLC pathway was severely influenced in the miR-139 inhibitor-transfected IND-damaged GES-1 cells. As illustrated in Figure 6(B and C), the CXCR4, CXCL12 mRNA and protein expression as well as p-PLC, p-PKC, Rho A, MLCK, and p-MLC protein expression levels were distinctly raised in the miR-139 inhibitor-transfected IND-damaged GES-1 cells compared to the IND or inhibitor NC group. In addition, pretreatment with TA (12.5 μM) used alone or combined with the miR-139 inhibitor further elevated CXCR4, CXCL12 mRNA and protein expression and p-PLC, p-PKC, Rho A, MLCK and p-MLC protein expression compared to the IND group or inhibitor NC group (*p*** **<** **0.01, respectively).

**Figure 7.**
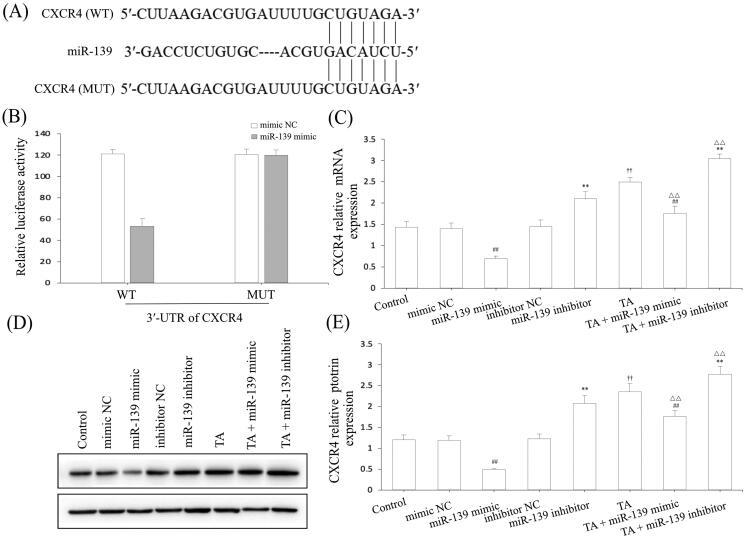
CXCR4 was a direct target of miR-139. (A) miR-139 binding site in the 3′-UTR of CXCR4 predicted by bioinformatics analysis. (B) The luciferase activity assay. (C) CXCR4 mRNA expression. (D) and (E) CXCR4 protein expression. The GES-1 cells were pretreated with TA used alone or combined with the miR-139 mimics and miR-139 inhibitor. The data are indicated as the mean ± SD (*n* = 5). ^#^*p* < 0.05, ^##^*p* < 0.01 compared to the mimic NC group; *p < 0.05, **p < 0.01 compared to the inhibitor NC group; ^†^*p* < 0.05, ^††^*p* < 0.01 compared to the control group; ^△^*p* < 0.05, ^△△^*p* < 0.01 compared to the TA group.

Based on the above data, we found that miR-139 had a negative regulatory effect on CXCR4 of IND-damaged GES-1 cells. Notwithstanding, whether CXCR4 was the underlying target gene of miR-139 was still unknown. To demonstrate this, we forecasted it using bioinformatics analysis on the online database. As demonstrated in Figure 7(A), CXCR4 was forecasted to be a presumptive target gene of miR-139. To further demonstrate this point, the luciferase reporter assay was executed after transfected with WT or MUT 3'-UTR sequence of CXCR4 combined with mimic NC or miR-139 mimic in GES-1 cells. The results indicated that transfection with miR-139 mimic apparently reduced the luciferase enzyme activity co-transfected with WT 3'-UTR of CXCR4, but caused no influence on luciferase enzyme activity in MUT 3'-UTR of CXCR4 (Figure 7(B)). Concurrently, we also discovered that the CXCR4 mRNA and protein expression levels were significantly suppressed by miR-139 mimic, and substantially improved by miR-139 inhibitor, TA used alone or combined with TA in GES-1 cells (Figure 7(C–E)). Consequently, CXCR4 is the target gene of miR-139, and the knockdown of miR-139 or pretreatment with TA effectively activated the CXCR4/CXCL12/PLC/PKC/Rho A/MLC pathway.

### CXCR4 knockdown attenuated the cytoprotection and migration of TA against IND-damaged GES-1 cells

**Figure 8.**
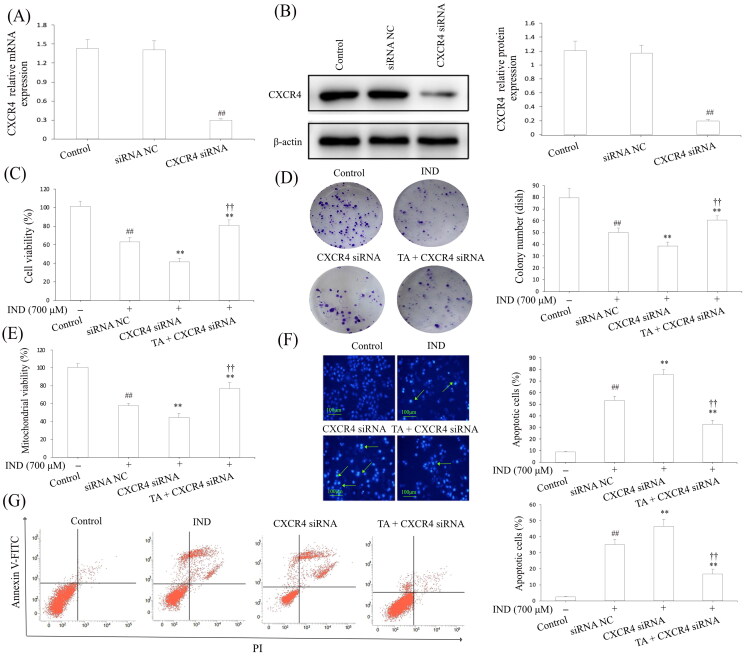
CXCR4 knockdown attenuated the cytoprotection and migration of TA against IND-damaged GES-1 cells. (A) CXCR4 mRNA expression. (B) CXCR4 protein expression. (C) Cell viability by MTT analysis. (D) Cell proliferation by colony formation analysis. (E) Mitochondrial viability by flow cytometry analysis. (F) Apoptotic cells by Hoechst-staining analysis. (G) Apoptotic cells by flow cytometry analysis. The GES-1 and IND-damaged GES-1 cells were transfected with CXCR4 siRNA. The data are indicated as the mean ± SD (*n* = 5). ^#^*p* < 0.05, ^##^*p* < 0.01 compared to the control group; *p < 0.05, **p < 0.01 compared to the IND group; ^†^*p* < 0.05, ^††^*p* < 0.01 compared to the CXCR4 siRNA group.

**Figure 9.**
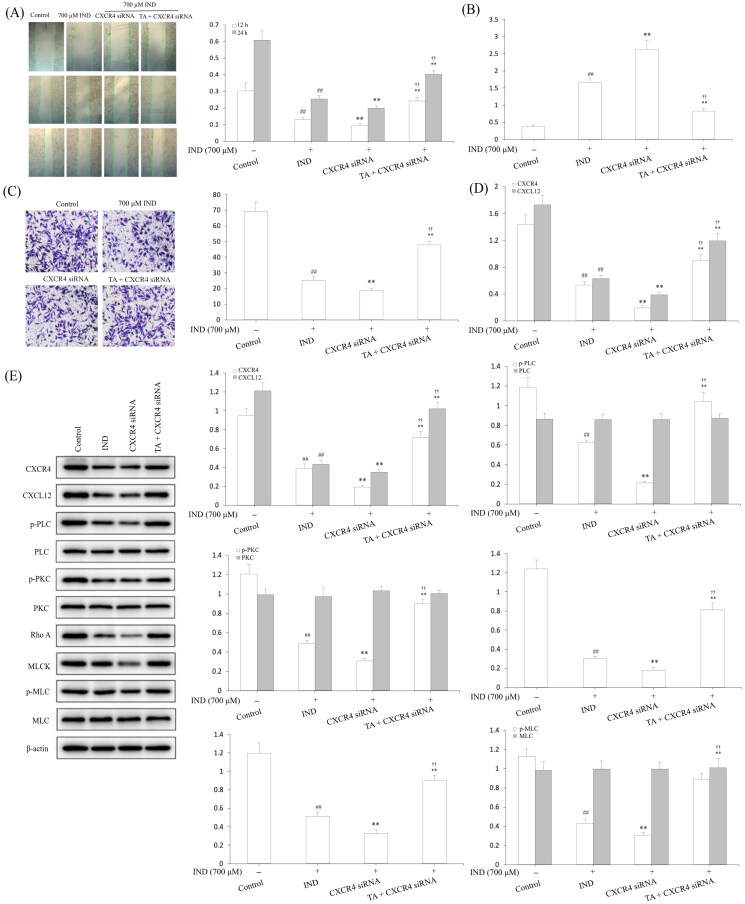
CXCR4 knockdown suppressed the migration and the CXCR4/CXCL12/PLC/PKC/Rho A/MLC pathway activation of TA on IND-damaged GES-1 cells. (A) Cell migration by wound healing assay. (B) Cell migration by Transwell assay. (C) miR-139 mRNA expression. (D) CXCR4 and CXCL12 mRNA expression. (E) Related protein expression of CXCR4/CXCL12/PLC/PKC/Rho a/MLC pathway. The GES-1 and IND-damaged GES-1 cells were transfected with CXCR4 siRNA. The data are indicated as the mean ± SD (*n* = 5). ^#^*p* < 0.05, ^##^*p* < 0.01 compared to the control group; *p < 0.05, **p < 0.01 compared to the IND group; ^†^*p* < 0.05, ^††^*p* < 0.01 compared to the CXCR4 siRNA group.

To intensify the evidence linking the cytoprotection and migration of TA are related to CXCR4, we transfected CXCR4 siRNA in GES-1 cells to knockdown the CXCR4 expression. Figure 8(A and B) revealed that the mRNA and protein expression of CXCR4 in GES-1 cells were remarkably reduced by CXCR4 siRNA transfection compared with the siRNA NC and control groups (*p*** **<** **0.01, respectively). In IND-damaged GES-1 cells, transfection with CXCR4 siRNA obviously reduced GES-1 cell migration, proliferation, mitochondrial viabilities, facilitated apoptosis and miR-139 expression compared to the IND group (*p*** **<** **0.01, respectively). After pretreatment with TA, the suppressed cell migration, proliferation, mitochondrial viabilities, elevated apoptosis and miR-139 expression was significantly reversed in IND-damaged GES-1 cells compared to the CXCR4 siRNA group (*p*** **<** **0.01, respectively, Figures 8(C–G) and 9(A–C)). Furthermore, qRT-PCR and western blotting results also displayed that the CXCR4 knockdown apparently weakened the CXCR4, CXCL12 mRNA and protein expression and p-PLC, p-PKC, Rho A, MLCK and p-MLC protein expression levels compared to the IND group (*p*** **<** **0.01, respectively, Figure 9(D–E)). In comparison, TA pretreatment efficiently redressed the abnormal changes in the above-mentioned indexes of the IND-damaged GES-1 cells after transfected with CXCR4 siRNA (*p*** **<** **0.01, respectively, Figure 9(D–E)), which was also consistent with the gastroprotection of TA and promoting migration effects on the IND- damaged GES-1 cells (Figure 5(C)). In brief, CXCR4 knockdown results added to the evidence that CXCR4 siRNA and TA pretreatment could generate an antagonistic effect on IND-damaged GES-1 cells. The present data suggested that CXCR4 was positively drawn into TA-mediated cytoprotection and promoting migration.

### TA administration implemented the gastroprotective effect on IND-induced gastric damage rats

**Figure 10.**
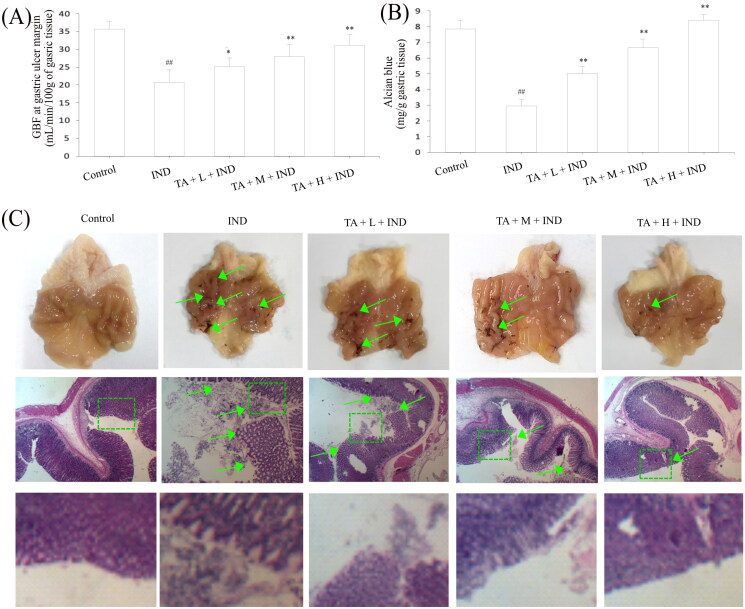
TA administration implemented a gastroprotective effect on IND-induced gastric damage rats. (A) GBF at gastric ulcer margin. (B) Mucus level in the gastric mucosa. (C) Macroscopic and microscopic photos of representative gastric mucosa. The data are indicated as the mean ± SD (*n* = 10). ^#^*p* < 0.05, ^##^*p* < 0.01 compared to the control group; *p < 0.05, **p < 0.01 compared to the IND group. Original magnification ×200 and ×400.

Ultimately, the gastroprotection of TA on IND-induced gastric mucosal lesions in rats was assessed. As illustrated in Figure 10(A), the GBF of the ulcer margin in the IND group decreased to 39.06% compared to the control group (*p*** **<** **0.01). Pretreatment with TA (1, 2, and 4 mg/kg) dramatically elevated the GBF of ulcer margin by 21.59%, 34.70% and 49.48%, respectively, compared with the IND group (*p*** **<** **0.05 or *p*** **<** **0.01, respectively). Unusually changed activities of oxidant and antioxidant enzymes and the cytokine contents were found in the IND group. Relative to the control group, decreased antioxidant activities [T-AOC (57.03%, *p*** **<** **0.01), GSH-Px (14.76%, *p*** **<** **0.01), SOD (24.04%, *p*** **<** **0.01), CAT (59.62%, *p*** **<** **0.01)] and anti-inflammatory factor contents [IL-4 (58.67%, *p*** **<** **0.01) and IL-10 (62.03%, *p*** **<** **0.01)], elevated MDA production (133.76%, *p*** **<** **0.01) and pro-inflammatory factor contents [TNF-α (52.34%, *p*** **<** **0.01), IL-1β (252.72%, *p*** **<** **0.01) and IL-6 (51.91%, *p*** **<** **0.01)] were found in the IND-induced rats. Pretreatment with TA (1, 2, and 4 mg/kg) markedly attenuated all aforementioned abnormalities of oxidative stress and cytokine levels (*p*** **<** **0.05 and *p*** **<** **0.01, respectively) compared to the IND rats (Tables 2 and 3).

**Table 2.** Effects of TA on antioxidant assay in the serum of IND-induced gastric damage rats.

Experimental group	T-AOC (U/mL)	GSH-Px (U/mL)	SOD (U/mL)	CAT (U/mL)	XOD (U/mL)	MDA (U/mL)
Control	29.39 ± 1.78	1701 ± 93	227.1 ± 18.5	0.52 ± 0.04	6.18 ± 0.67	2.34 ± 0.21
IND	12.63 ± 1.24^##^	1450 ± 35^##^	172.5 ± 13.7^##^	0.21 ± 0.02^##^	12.25 ± 1.19^##^	5.47 ± 0.42^##^
TA-L + IND	16.77 ± 1.19**	1513 ± 34*	199.6 ± 16.0*	0.26 ± 0.03*	10.58 ± 1.07*	4.85 ± 0.32*
TA-M + IND	21.88 ± 2.02**	1580 ± 47**	206.9 ± 17.8**	0.35 ± 0.04**	9.25 ± 0.98**	4.09 ± 0.29**
TA-H + IND	26.01 ± 2.13**	1673 ± 55**	215.4 ± 19.1**	0.46 ± 0.03**	8.13 ± 0.86**	3.24 ± 0.27**

The data were indicated as the mean ± SD (*n* = 10). ^#^*p* < 0.05, ^##^*p* < 0.01 compared to the control group; *p < 0.05, **p < 0.01 compared to the IND group.

**Table 3.** Effects of TA on cytokines in the gastric mucosal tissues of IND-induced gastric damage rats.

Experimental group	TNF-α (pg/mg)	IL-1β (pg/mg)	IL-6 (pg/mg)	IL-4 (pg/mg)	IL-10 (pg/mg)
Control	13.47 ± 0.96	9.01 ± 0.82	7.05 ± 0.56	46.17 ± 3.56	71.71 ± 5.82
IND	20.52 ± 1.77^##^	31.78 ± 2.57^##^	10.71 ± 1.09^##^	19.62 ± 1.58^##^	27.23 ± 2.32^##^
TA-L + IND	18.03 ± 1.32*	27.57 ± 2.23*	9.13 ± 0.87*	22.28 ± 1.92*	31.29 ± 2.87*
TA-M + IND	16.52 ± 1.24**	20.34 ± 1.82**	8.35 ± 0.76**	30.14 ± 2.09**	42.69 ± 3.78**
TA-H + IND	14.71 ± 1.05**	13.67 ± 1.07**	7.90 ± 0.65**	41.91 ± 2.94**	64.42 ± 4.07**

The data were indicated as the mean ± SD (*n* = 10). ^#^*p* < 0.05, ^##^*p* < 0.01 compared to the control group; *p < 0.05, **p < 0.01 compared to the IND group.

Gastric mucus, bicarbonate and phospholipids constitute the first protective barrier of gastric mucosa, which effectively depresses the harmful effects of invasive factors on gastric mucosa (He et al. 2020). Hence, the amount of gastric mucus adhered to is an important indicator to evaluate the effect of drugs on gastric mucosa. As indicated in Figure 10(B), the experimental rats administered IND by gavage caused remarkable exhaustion of gastric mucus compared to the control group (*p*** **<** **0.01). TA administration (1, 2, and 4 mg/kg) effectively suppressed the exhaustion of gastric mucus and raised the amount of gastric mucus adhered by 70.50%, 125.64% and 184.87%, respectively, compared to the IND group (*p*** **<** **0.01, respectively). As is generally known, the abnormal gastric acid secretion is an essential factor leading to gastric mucosal layer lesions. Thus, suppressing gastric acid secretion has become one of the important means to treat gastric mucosal injury (He et al. 2017b). In the current experiment, the volume and total acidity of the gastric effluents apparently elevated, and the gastric pH markedly reduced in the IND group compared to the control group (*p*** **<** **0.01, respectively). Nevertheless, pretreatment with TA (1, 2, and 4 mg/kg) prominently dampened the volume by 13.48%, 44.94%, 65.17%, respectively, and total acidity of the gastric effluents by 17.06%, 48.82%, 71.06%, respectively, and raised the gastric pH by 17.77%, 42.13% and 68.53%, respectively, compared to the IND group (*p*** **<** **0.05 or *p*** **<** **0.01, respectively, Table 4).

**Table 4.** Effects of TA on the gastric ulcer and gastric acid secretions of IND-induced gastric damage rats.

Experimental group	Ulcer area (mm^2^)	Ulcer inhibitor (%)	Gastric juice volume (mL)	pH (Units)	[H^+^] (mEq/L 4h)
Control	0.00 ± 0.00	----	0.18 ± 0.02	3.49 ± 0.28	1.76 ± 0.14
IND	56.14 ± 4.39^##^	0.00 ± 0.00	0.89 ± 0.08^##^	1.97 ± 0.19^##^	8.50 ± 0.81^##^
TA-L + IND	29.27 ± 2.19**	47.72 ± 4.35**	0.77 ± 0.07*	2.32 ± 0.20*	7.05 ± 0.72*
TA-M + IND	17.59 ± 1.28**	68.49 ± 3.64**	0.49 ± 0.06**	2.80 ± 0.23**	4.35 ± 0.43**
TA-H + IND	7.67 ± 0.75**	86.25 ± 1.87**	0.31 ± 0.03**	3.32 ± 0.26**	2.46 ± 0.22**

The data were indicated as the mean ± SD (*n* = 10). ^#^*p* < 0.05, ^##^*p* < 0.01 compared to the control group; *p < 0.05, **p < 0.01 compared to the IND group.

Modern research has revealed that the gastric mucosa has a “gastric mucosal defense barrier” system composed of defense factors: mucus bicarbonate barrier, mucosal barrier, epidermal growth factor, prostaglandin, etc., which coordinate with each other to resist the damage of attack factors to the gastric mucosa. However, when the above barrier was damaged or the secretion of protective factors was insufficient, the protective effect of gastric mucosa would be weakened, and gastric ulcer would occur under the irritant actions of attack factors such as gastric acid and pepsin (Yandrapu and Sarosiek 2015). In the current experiment, the ulcer area of IND group had topped out at 56.14 ± 4.39 mm^2^. After pretreated with TA (1, 2, and 4 mg/kg), their ulcer areas fell to 29.27 ± 2.19, 17.59 ± 1.28, 7.67 ± 0.75 mm^2^, respectively, and the ulcer inhibition rates were 47.72 ± 4.35, 68.49 ± 3.64, 86.25 ± 1.87%, respectively, and there were dramatic improvements compared with the IND group (*p*** **<** **0.01, respectively, Figure 10(C) and Table 4). Gastric mucosal histomorphology by H&E staining further revealed that the layers of gastric mucosa were clear, the epithelium was complete, the glands were arranged orderly, and there was no congestion and erosion in the control group; on the contrary, hyperemia, dilation and erosive bleeding of gastric mucosa, epithelial cell abscission, disordered arrangement of glands and inflammatory cell infiltration were observed, and its gastric mucosal damage scores of haemorrhages, oedema, epithelial cell loss and inflammatory cell infiltration in the IND model reached 3.54 ± 0.58, 3.28 ± 0.56, 2.65 ± 0.40, and 2.60 ± 0.47, respectively, and there were significant suppressions compared with the control group (*p*** **<** **0.01, respectively). TA (1, 2, and 4 mg/kg) safeguarded gastric tissue against hemorrhagic lesions, obviously alleviated the gastric mucosal haemorrhages, oedema, epithelial cell loss and inflammatory cell infiltration, its improvement ratios were 19.68%, 53.99% and 82.98% respectively; 16.81%, 58.55%, 86.96%; 42.31%, 70.83%, 87.82%, and 21.15%, 70.61%, 88.53% compared to with the IND group (*p*** **<** **0.05 or *p*** **<** **0.01, respectively, Figure 10(C) and Table 5.

**Table 5.** Effects of TA on the gastric mucosal lesion score of IND-induced gastric damage rats.

Experimental group	Hemorrhage score	Oedema score	Epithelial cell loss score	Inflammatory cell score	Total score
Control	0.00 ± 0.00	0.00 ± 0.00	0.11 ± 0.02	0.00 ± 0.00	0.11 ± 0.02
IND	3.76 ± 0.38^##^	3.45 ± 0.34^##^	3.12 ± 0.27^##^	2.79 ± 0.35^##^	13.13 ± 0.87^##^
TA-L + IND	3.02 ± 0.40*	2.87 ± 0.26*	1.80 ± 0.12**	2.20 ± 0.26*	9.90 ± 0.63**
TA-M + IND	1.73 ± 0.15**	1.43 ± 0.17**	0.91 ± 0.07**	0.82 ± 0.08**	4.89 ± 0.21**
TA-H + IND	0.64 ± 0.06**	0.45 ± 0.04**	0.38 ± 0.03**	0.32 ± 0.03**	1.79 ± 0.05**

The data were indicated as the mean ± SD (*n* = 10). ^#^*p* < 0.05, ^##^*p* < 0.01 compared to the control group; *p < 0.05, **p < 0.01 compared to the IND group.

### TA suppressed miR-139 expression and activated the CXCR4/CXCL12/PLC/PKC/Rho a/MLC pathway in IND-induced gastric damage rats

**Figure 11.**
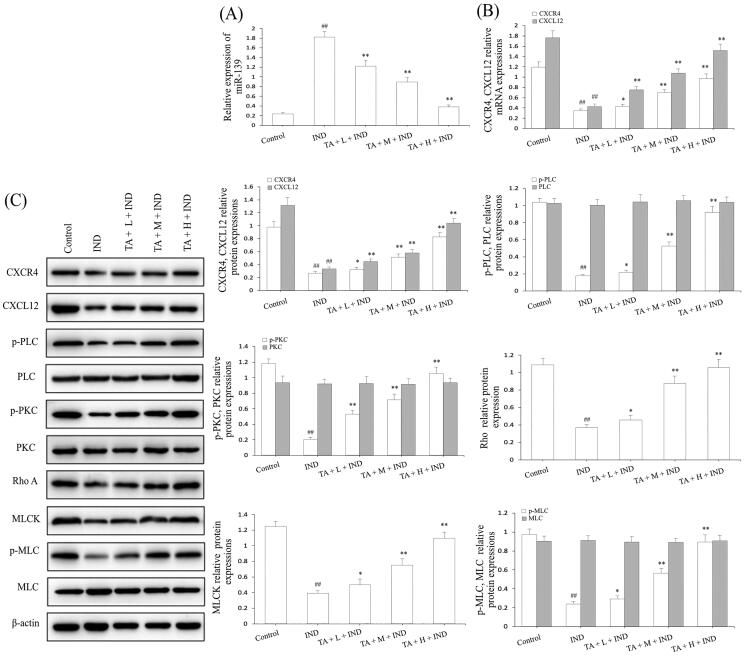
TA restrained miR-139 expression and activated the CXCR4/CXCL12/PLC/PKC/Rho A/MLC pathway in IND-induced gastric damage rats. (A) miR-139 mRNA expression. (B) CXCR4 and CXCL12 mRNA expression. (C) Related protein expression of the CXCR4/CXCL12/PLC/PKC/Rho a/MLC pathway. The data are indicated as the means ± SD (*n* = 5). ^#^*p* < 0.05, ^##^*p* < 0.01 compared to the control group; *p < 0.05, **p < 0.01 compared to the IND group.

After amply verifying the gastroprotective effect of TA on IND-induced gastric damage rats, we next detected its underlying mechanism. Firstly, the miR-139 mRNA expression of gastric tissue was analyzed by qRT-PCR. The miR-139 mRNA expression of IND-damaged gastric tissue in rats prominently raised compared to the control group (*p*** **<** **0.01); On the contrary, TA (1, 2, and 4 mg/kg) dramatically repressed miR-139 mRNA expression compared to the IND group (*p*** **<** **0.01, respectively, Figure 11(A)). The current experimental data were coincident with the *in vitro* results.

Subsequently, we analyzed the related protein expression levels of the CXCR4/CXCL12/PLC/PKC/Rho A/MLC pathway. The qRT-PCR and western blotting data of rat gastric mucosa indicated that CXCR4 and CXCL12 mRNA and protein expression levels were dramatically reduced (*p*** **<** **0.01, respectively); TA pretreatment prominently elevated CXCR4 and CXCL12 mRNA and protein expression by 22.06%, 99.89%, 178.86%, 75.98%, 152.89%, 256.14%, and 22.57%, 95.66%, 214.95%, 34.77%, 75.23%, 214.90% (*p*** **<** **0.05 or *p*** **<** **0.01, respectively, Figure 11(B and C)). Moreover, their downstream p-PLC, p-PKC, Rho A, MLCK, and p-MLC protein expression of the IND group was markedly depressed compared to the control group (*p*** **<** **0.01, respectively). After pretreatment of TA, the abnormal protein expression of p-PLC, p-PKC, Rho A, MLCK, and p-MLC was obviously reversed compared to the IND group (*p*** **<** **0.05, *p*** **<** **0.01, respectively, Figure 11(C)). The above-mentioned results demonstrated that miR-139 and its mediated downstream CXCR4/CXCL12/PLC/PKC/Rho A/MLC pathway participated in TA promoting the proliferation and migration of gastric mucosal epithelial cells, and then ameliorated the damaged gastric mucosal repair and healing process in the IND-induced gastric damage rats, which was coincident with the data of *in vitro* experiments.

## Discussion

Mugua is the dry and nearly mature fruit of *Chaenomeles speciosa*, a crop that has unique medicinal value and nutritional healthcare function, which also is a healthy food with many nutrients. In clinical practice, it is mainly used to treat various rheumatic diseases (China Pharmacopoeia Committee 2020); at the same time, it is also a high-quality raw material for processing canned Mugua, fruit pulp, wine, beverages, and other foods, skin care products, and bath products. There is a folk saying that “apricot one benefit, pear two benefits and Mugua 100 benefits”, so Mugua is also known as “the fruit of 100 benefits” (He et al. 2020). In China, Tujia doctors and residents soak Mugua in corn wine to make Mugua wine or pick immature Mugua to make appetizers (Mugua pickles, Mugua sauce, and Mugua vinegar) and snacks (fruit juice, preserved fruits, preserves, etc.) to resist rheumatic diseases and gastrointestinal injuries that may be caused by the long rainy and humid mountain environment and the habit of eating spicy and pickled/smoked meat. Through the follow-up survey in recent 10 years, we did find that the probabilities of suffering from these two diseases were much lower than that in non-Mugua producing areas and people who do not eat Mugua (Wang et al. 2015). Inspired by these, we successfully screened triterpenoids from *C. speciosa* fruits, and demonstrated that they have a good protective effect on IND-damaged rat gastric mucosal epithelial cells, GES-1 cells and IND- and ethanol-damaged gastric mucosal injury in rats and mice. Triterpenoids from *C. speciosa* fruits were pentacyclic triterpenoids, which were divided into oleanane-type triterpenoids (oleanolic acid, maslinic acid), lupane-type triterpenoids (betulinic acid) and ursane-type triterpenoids (ursolic acid, 3-*O*-acetyl ursolic acid, 3-*O*-acetyl pomolic acid, and TA) (Qin et al. 2015, 2016a, 2016b; Shi et al. 2016; He et al. 2017a, 2017b, 2019a, 2019b, 2021; Zhang et al. 2020). To compare the differences in their gastric protective effects and further explore their possible mechanism, we implemented the present study.

In the study, our results indicated that TA was the most gastroprotective component of the pentacyclic triterpenoids (oleanolic acid, maslinic acid, betulinic acid, ursolic acid, 3-*O*-acetyl ursolic acid, 3-*O*-acetyl pomolic acid, and TA) against IND-damaged GES-1 cells. It might advance IND-damaged GES-1 cell proliferation and migration, repress apoptosis, ameliorate IND-damaged rat GBF of ulcer margin, ulcer area and inhibition rate, reverse the activities and levels of the redox system and cytokine, elevate the amount of adherent gastric mucus, weaken the volume and total acidity of the gastric effluents, raise the gastric pH, mitigate gastric mucosal bleeding, submucosal edema, epithelial cell loss, suppress inflammatory cell infiltration and upregulate mitochondrial viability, CXCR4 and CXCL12 mRNA and protein expression, p-PLC, p-PKC, Rho A, MLCK, and p-MLC protein expression. Our current study demonstrated that the gastroprotective effect of TA was closely correlated with suppressing miR-139 expression, activating the activated CXCR4/CXCL12/PLC/PKC/Rho A/MLC pathway, which in turn promoted the proliferation and migration of gastric mucosal epithelial cells, and ameliorated the damaged gastric mucosal repair and healing.

Recently, miR-139 has been widely investigated in many human diseases. For example, osteoarthritis, bronchial asthma, myocardial injury, breast cancer, etc. (Cheng et al. 2021). Zhang et al. (2018) discovered that the expression levels of miR-139 in breast cancer cell lines (MCF-7, BT-474, SKBR3, MDA-MB-231) and the patient’s breast cancer tissues were downregulated. Overexpression of miR-139 prominently repressed CXCR4 and CXCL12 expression in MDA-MB-231 cells and suppressed cell metastasis *in vivo*, and confirmed that miR-139 restrained the CXCR4/CXCL12 signal axis to reduce the metastasis of breast cancer. Furthermore, Baskara-Yhuellou and Tost (2020) also verified that the overexpression of miR-139 could suppress the proliferation of bronchial smooth muscle cells, accelerate apoptosis, and ameliorate airway remodelling and hyperresponsiveness in asthma. Interestingly, we first discovered that the mRNA expression of miR-139 was evidently raised, and the mRNA and protein expression of CXCR4 and CXCL12 were obviously depleted in gastric ulcer patients, but the aforementioned expressions were effectively reversed with the healing of gastric mucosa (Rodríguez et al. 2003). In the current study, the mRNA expression of miR-139 in the IND-damaged GES-1 cells and rat gastric tissues noticeably manifested the increased tendency compared to the GES-1 cells and rats, and pretreatment with TA markedly repressed its expression. Furthermore, the overexpression of miR-139 attributed to miR-139 mimic transfection further exacerbated the cell damage induced by IND, which was related to cell migration and proliferation suppression, mitochondrial viability depression and apoptosis elevation. In contrast, the knockdown of miR-139 by transfecting with miR-139 inhibitor or pretreatment with TA used alone or in combination with miR-139 inhibitor could notably accelerate cell migration and proliferation, enhance mitochondrial viability and restrain apoptosis. The changes of miR-139 of the gastric tissues in the IND-damaged rats were also similar to the miR-139 expression of IND-damaged GES-1 cells.

As is well known, miRs are involved in biological processes such as growth and development, differentiation, signal transduction, cell migration, proliferation and apoptosis, which play a vital role in a variety of physiological and pathological processes (including trauma repair, tumours) by regulating its target genes (Cui et al. 2017). Therefore, it was necessary to further study the target genes regulated by miR-139 in our experiment. Therefore, we forecasted and verified it by using bioinformatics prediction analysis and luciferase reporter gene assay in the current study. The results demonstrated that miR-139 might directly combine with the 3'-UTR region of CXCR4 mRNA, and for this reason, CXCR4 might be identified as a target gene of miR-139. CXCR4 is a cognate seven transmembrane G protein-coupled receptor involved in regulating the biological functions of cell survival, migration, proliferation, chemotaxis, apoptosis and differentiation, and strengthening angiogenesis, wound reepithelialization in targeted diseases (Smith et al. 2005; Chen et al. 2021). Guo et al. (2015) indicated that the CXCL12 expression levels at the wound edges were dramatically elevated after injury, and CXCR4 expression was also substantially promoted in proliferating epithelial cells. In addition, obstructing the CXCR4 and CXCL12 led to remarkable repression in epidermal cell migration toward SDF-1 *in vitro* and postponed wound healing *in vivo*. After gastric mucosal injury, CXCR4 and its ligand CXCL12 constituted the chemokine-chemokine receptor axis in epithelial cell homing, and the activation of CXCR4 might play an important role in migrating pre-existing or externally transplanted epithelial cells to the damaged site, and then repairing damaged gastric mucosal tissue and promoting wound healing. Moreover, *in vitro* chemotaxis experiments demonstrated that epithelial cells overexpressing CXCR4 could promote the migration to CXCL12 (Rao et al. 2001; Guo et al. 2015; Chen et al. 2021; Wang et al. 2021). Therefore, CXCR4 and its ligand CXCL12 play a crucial role in the healing of damaged gastric mucosa. In the present study, the mRNA and protein expression levels of CXCR4 and CXCL12 were obviously repressed in IND-damaged GES-1 cells and rat gastric tissues, and the negative or positive regulatory relationship between them and miR-139 was further confirmed by miR-139 mimic or miR-139 inhibitor experiment in IND-damaged GES-1 cells. Moreover, transfection with CXCR4 siRNA might obviously reduce GES-1 cell migration and proliferation, facilitated apoptosis and miR-139 expression. By contrast, the knockdown of miR-139 by transfecting with miR-139 inhibitor or pretreatment with TA used alone or in combination with miR-139 mimic, miR-139 mimic inhibitor or CXCR4 siRNA in the IND-damaged GES-1 cells significantly reversed the reduced CXCR4 and CXCL12 mRNA and protein expression levels. Pretreatment with TA markedly elevated the CXCR4 and CXCL12 mRNA and protein expression levels of the gastric tissues in IND-induced gastric damage rats. Based on the above clarifications, we concluded that miR-139 negatively regulated its downstream CXCR4 target. The data demonstrated that the gastroprotective effect of TA on IND-damaged GES-1 and rats was closely related with restraining miR-139 expression and activating CXCR4/CXCL12 signal axis.

It has been well-documented that the CXCR4/CXCL12/PLC/PKC/Rho A/MLC pathway plays a crucial role in repairing gastric mucosal lesions, and authenticated that the CXCR4/CXCL12 signal axis facilitates the epithelial cell migration and the formation and repair of mucosal barrier are not an independent process, which needs the participation of a series of downstream signal pathways, such as the PLC/PKC/Rho A/MLC pathway (Shimada and Terano 1998; Kountouras et al. 2005; Mi et al. 2021). On the one hand, after CXCR4/CXCL12 is activated, it might quickly activate G protein-coupled receptors on the surface of epithelial cells, trigger PLC/PKC signal cascade transmission through relevant adaptor molecules (such as Shc, PLC, etc.), activate Rho GTPase family members (for example, RhoA, Rac1 and Cdc42), induce the rapid phosphorylation of cytoskeleton molecule β-catenin, and then lead to the separation of E-cadherin/catenin complex from actin cytoskeleton and the instability of cell-cell adhesion connection, thus accelerate the phosphorylation of amino acid residues of tight junction proteins (such as claudin, occludin, JAM, ZO, cingulin, etc.), activate the integrin signal transduction, boost the focal adhesion kinase phosphorylation, sabotage cell matrix adhesion, alter cytoskeleton, reinforces MLCK activity, accelerate MLC phosphorylation and then facilitate epithelial cell migration (Rao et al. 2001; Akimoto et al. 2005; Hwang et al. 2012; Carrasco-Pozo et al. 2016; Amoozadeh et al. 2018; Feng et al. 2022). When the epithelial cells migrated to the injured site, activated CXCR4/CXCL12 could, on the other hand, stimulate epithelial cell repolarization, reconstitute cell-cell connection and cell-matrix adhesion, advance epithelial cell proliferation and differentiation, reconstruct mucosal barrier, and then accelerate the healing of damaged gastric mucosa through activating the PLC/PKC/Rho A/MLC pathway (Rathinam et al. 2011; Aihara et al. 2018). All of these demonstrated that the healing of damaged gastric mucosa was closely related to activating the PLC/PKC/Rho A/MLC pathway mediated by the CXCR4/CXCL12 signal axis. In our study, the obviously abated p-PLC, p-PKC, Rho A, MLCK, and p-MLC protein expression was found in IND-damaged GES-1 cells and rat gastric tissues, and the aforementioned expression levels in the miR-139 mimic or CXCR4 siRNA-transfected IND-damaged GES-1 cells were further worsened. While transfection with miR-139 inhibitor might effectively keep these changes down. Similarly, whether TA used alone, combined with miR-139 mimic or CXCR4 siRNA transfection in the IND-damaged GES-1 cells might successfully reverse the anomalous protein expression of p-PLC, p-PKC, Rho A, MLCK, and p-MLC; by contrast, TA used alone or in combined with miR-139 inhibitor in the IND-damaged GES-1 cells, further elevated the p-PLC, p-PKC, Rho A, MLCK, and p-MLC protein expression levels. All of these outcomes were in keeping with the enhancement of migration, proliferation in IND-damaged GES-1 cells and the GBF, gastric ulcer, gastric acid secretions, gastric mucosal damage scores, and histopathological changes of gastric mucous tissue in IND-damaged rats. These data indicated the gastroprotective effect of TA on IND-damaged GES-1 cells and rats by activating the CXCR4/CXCL12/PLC/PKC/Rho A/MLC pathway.

## Conclusions

**Figure 12.**
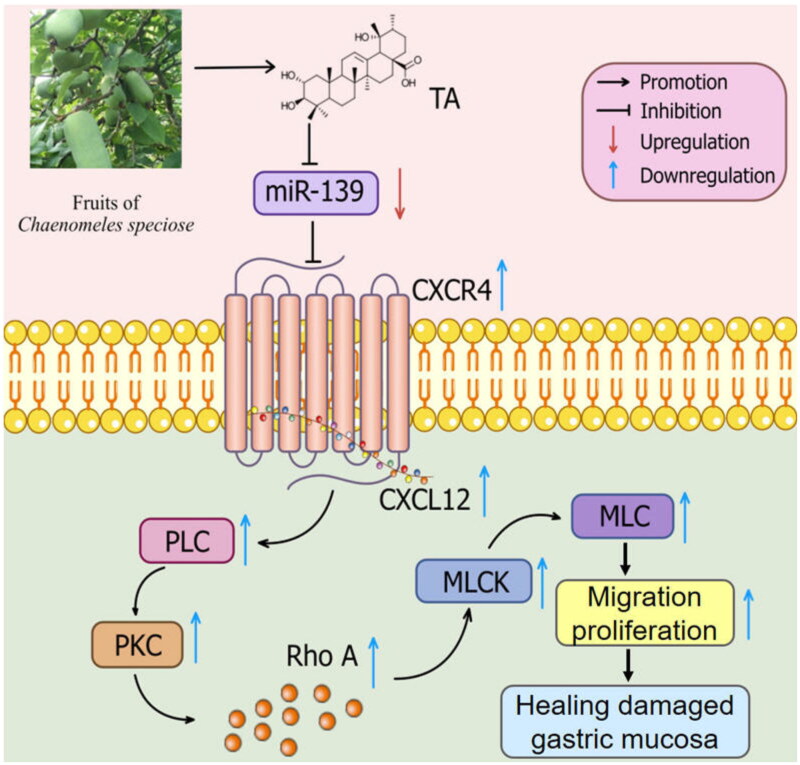
Potential molecular mechanism of TA-exerted gastroprotective effect on IND-induced gastric mucosal lesion.

The present study authenticated for the first time that TA could mediate protection characteristics against IND-induced gastric mucosal injury *in vitro* and *in vivo*, which was tightly related to restraining miR-139 expression and activating the CXCR4/CXCL12/PLC/PKC/Rho A/MLC pathway, thereby enhancing epithelial cell migration and proliferation, reconstructing mucosal barrier, and then accelerating the healing of damaged gastric mucosa (Figure 12). These data furnished new concepts for elucidating the potential mechanism of the gastroprotective effect of TA and show promise in using this agent as a clinical drug candidate for gastric mucosal lesion treatment.
